# Endobiogenic Biology of Function Indices in a Cohort of Kidney Transplant Recipients

**DOI:** 10.3390/medicina60061016

**Published:** 2024-06-20

**Authors:** Ernesta Mačionienė, Danielius Serapinas, Marius Miglinas

**Affiliations:** 1 Gastroenterology, Nephrourology and Surgery Clinic, Faculty of Medicine, Vilnius University, LT03101 Vilnius, Lithuania; marius.miglinas@santa.lt; 2 Department of Family Medicine, Medical Academy, Lithuanian University of Health Sciences, LT44307 Kaunas, Lithuania; danielius.serapinas@lsmuni.lt

**Keywords:** kidney transplant, transplant rejection, endobiogeny, biology of function index

## Abstract

*Background and Objectives*: Endobiogeny is a global systems approach to human biology based on the concept that the endocrine system manages the metabolism. Biology of function (BoF) indices are diagnostic tools in endobiogenic medicine that reflect the action of the endocrine system on the cells and the metabolic activity of an organism. Kidney transplant recipients are a very specific patient population due to their constant use of immunosuppressive agents such as steroids and anamnesis of chronic kidney disease. The aim of this study was to assess the tendencies of endobiogenic BoF indices in a kidney transplant recipient population and to determine the relationship between BoF index values and histology-proven kidney transplant rejection. *Materials and Methods*: A total of 117 kidney transplant recipients undergoing surveillance or indication allograft biopsy were included in this study. Endobiogenic BoF indices were calculated from complete blood count tests taken before the kidney biopsy. Histology samples were evaluated by an experienced pathologist according to the Banff classification system. Clinical and follow-up data were collected from an electronic patient medical record system. *Results*: Overall, <35% of the patients had BoF index values assumed to be normal, according to the general population data. Additionally, >50% of the patients had lower-than-normal adaptation, leucocyte mobilization, genital, and adjusted genital ratio indices, while the Cata-Ana, genito-thyroid ratio, adrenal gland, and cortisol indices were increased in >50% of the transplant recipients. The adaptation index was significantly higher in patients with biopsy-proven transplant rejection and demonstrated an AUC value of 0.649 (95%CI 0.540–0.759) for discriminating rejectors from patients without transplant rejection. *Conclusions*: Most of the kidney transplant recipients had abnormal BoF index values, reflecting increased corticotropic effects on their cells. The adaptation index distinguished patients with biopsy-proven transplant rejection from those without it.

## 1. Introduction

Kidney transplantation is one of the most effective methods of renal replacement therapy in patients with end-stage renal disease (ESRD) [1,2]. After receiving a kidney transplant, patients have to take combined immunosuppressive therapy to avoid allograft rejection and maintain the function of the transplanted kidney [3,4,5]. There are some risks associated with constant immunosuppression, such as various bacterial, fungal, or viral infections, opportunistic infections, cancer, and metabolic complications. Therefore, an optimal balance between over- and under-immunosuppression must be maintained [6,7,8,9,10].

In the context of kidney transplantation, there are several diagnostic methods and biomarkers to predict and diagnose kidney transplant rejection or to monitor transplant function [11]. Kidney transplant rejection can be suspected when non-specific biomarkers such as serum creatinine or proteinuria levels increase, and rejection is diagnosed through kidney biopsy when characteristic histological abnormalities are present according to the Banff classification criteria [12,13]. Donor-specific antibodies are a biomarker of antibody-mediated transplant rejection. Moreover, there are more biomarkers, such as donor-derived cell-free DNA and the urinary chemokines CXCL9 and CXCL10, but none of them are highly specific to rejection as they may be increased in cases of viral infections, other sources of inflammation, and so on [14,15,16,17,18]. There are some biomarkers that can be used to evaluate the level of immunosuppression, including the concentrations of serum calcineurin inhibitors, pharmacogenetic biomarkers, or torque teno virus levels, providing guidance for immunosuppression [19,20]. However, there are still challenges associated with maintaining an optimal level of immunosuppression in individual patients, prolonging graft survival as long as possible, and avoiding the complications of over-immunosuppression throughout the patient‘s life.

Endobiogeny is a global systems approach to human biology and is based on the concept that the endocrine system manages the metabolism, resulting in biomarkers that reflect the functional achievement of specific aspects of the metabolism [21]. In endobiogeny, biology of function (BoF) indices are used to assess and monitor the functional balance of various systems within the body [22]. Direct indices are calculated from basic blood test results (e.g., the complete blood count with its differential) and various serum tests (e.g., those for electrolytes or lactate dehydrogenase), while indirect indices are calculated using patented software (Pub. No. US 2016/0132655 A1) by indexing various biomarkers and direct indices against each other [23]. BoF indices reflect the activity of different endocrine axes (corticotropic, gonadotropic, thyreotropic, and somatotropic), and can be used as tools to understand the nature of the disease and implement a holistic approach for individual patients.

Kidney transplant recipients are a very specific population of patients due to their prolonged use of immunosuppressive agents, history of long-lasting chronic kidney disease and, in most cases, a history of dialysis therapy before kidney transplantation [24]. All of these factors undoubtedly affect the patient‘s endocrine system and immune response [25,26,27].

In the existing literature, certain BoF indices have been analyzed in patients with myocardial infarction, COVID-19 infection, or heart failure [28,29,30]; however, we could not find any data about BoF indices in a kidney transplant recipient population. Therefore, the aim of this study was to calculate some of the BoF indices in kidney transplant recipients, enabling the analysis of the relationship between the BoF indices and biopsy-proven transplant rejection.

## 2. Materials and Methods

### 2.1. Study Description and Patient Population

A total of 117 randomly selected kidney transplant recipients admitted to the nephrology unit at Vilnius University Hospital Santaros Klinikos in 2017–2023 for a transplant biopsy were included in this study. The patient clinical data were collected retrospectively from an electronic patient medical data system. The laboratory test data included the complete blood count, biochemistry, immunosuppressive agent serum concentrations before kidney biopsy, and the serum creatinine level data both before biopsy and at follow-up (1, 3, and 6 months after transplantation and the last known follow-up). Kidney biopsies were evaluated at one center by an experienced pathologist and reported using the Banff scheme, applying the most up-to-date criteria at the time of reporting [12]. For further analysis, the biopsies were divided into three distinct groups: normal histology, rejection (antibody-mediated rejection (ABMR), T-cell-mediated rejection (TCMR), or mixed rejection), and other histology (global glomerulosclerosis, recurrent glomerulonephritis, thrombotic microangiopathy without rejection, calcineurin inhibitor toxicity-induced lesions, amyloidosis, interstitial nephritis, or BK virus nephritis).

The kidney transplant recipients received standard induction immunosuppressive therapy: basiliximab for moderate immunological risk and thymoglobulin for recipients with high immunological risk. Maintenance immunosuppressive therapy mostly consisted of tacrolimus or cyclosporine (the latter was used by earlier transplant patients), mycophenolate mofetil, and methylprednisolone. Patients with biopsy-proven rejection were treated according to the histological phenotype and severity. Briefly, TCMR episodes were treated with steroids, and severe clinical TCMR patients received thymoglobulin infusions. ABMR was mostly treated with plasmapheresis and intravenous immunoglobulins +/− rituximab. This study complied with all regulations, and informed consent was obtained from the participants. The experiments were conducted according to established ethical guidelines.

### 2.2. Statistical Analysis

Continuous variables are presented as mean ± standard deviation or median [interquartile range], according to the type of data. The normality of the quantitative data was tested using the Kolmogorov–Smirnov test. The Student’s *t*-test and the Wilcoxon Signed Rank test were applied to compare continuous variables with normal and skewed distributions, respectively. The Spearman’s rank correlation analysis was performed, and an unsupervised hierarchical clustering analysis was conducted to identify patient groups with similar BoF indices to explore their relationship with allograft outcomes. The ability of BoF indices to discriminate transplant rejection was analyzed using receiver operating characteristic (ROC) curves. A *p*-value less than 0.05 was considered statistically significant. Statistical analyses were performed with SPSS 29.0 (SPSS, Inc., Chicago, IL, USA).

### 2.3. BoF Indices

The BoF indices were calculated using formulas in the field of endobiogenic medicine [31]. A list of the indices that we calculated for kidney transplant recipients and a short explanation of these indices, according to the theory of endobiogeny, is provided as follows:

The Catabolism/Anabolism (Cata-Ana) index expresses the relative catabolic activity in relation to that of anabolic activity within the scheme of the global metabolism of the organism [31]. It is calculated from the ratios of neutrophils, lymphocytes, red blood cells (RBCs), and white blood cells (WBCs).

The adaptation index is the ratio of eosinophils to monocytes, which reflects the relative activity of adrenocorticotropic hormone (ACTH) on cortisol in relation to follicle-stimulating hormone (FSH) activity on estrogen during the adaptation response. Eosinophils vary similarly to ACTH and are inhibited by cortisol through sequestration in the spleen and lungs [32]. Monocytes vary similarly to FSH simulation and are inhibited by estrogens [33,34].

The cortisol index reflects the activity of cortisol. It does not directly indicate the concentration of cortisol in the blood but, instead, reflects the activity and effect of cortisol on cells. The formula for this index is based on the observation that cortisol increases erythrocytes, leukocytes, neutrophils, and monocytes, while diminishing lymphocytes, eosinophils, and the eosinophil/monocyte ratio [35,36,37,38]. The cortisol index formula includes the percentage of neutrophils, lymphocytes, eosinophils, and monocytes, as well as the absolute numbers of red blood cells and white blood cells (in thousands).

The adrenal gland index is an indirect index, calculated from the genito-thyroid ratio and genital ratio, which expresses the level of activity of the adrenal cortex [39].

The genital ratio index reflects the activity of androgens over estrogens and is calculated as the ratio of RBCs/(WBCs × 1000), according to the hypothesis that RBCs are a biomarker of the functional role of androgens in the metabolism and WBCs are a biomarker of the effects of estrogens on tissues [22].

The leukocyte mobilization index (LMI) evaluates the role of alpha-sympathetic activity in immediate adaptation [39]. A high LMI indicates that the alpha-sympathetic nervous system deliberates leukocytes more from splanchnic circulation than hepatic circulation. If the index is low, leukocytes are solicited more from perihepatic circulation than splanchnic circulation, indicating a tendency toward the de novo production of leukocytes from the bone marrow rather than liberation from the splanchnic reserve. The lower the index, the more dysfunctional the response to aggression. Its formula includes WBCs, platelets, hemoglobin, and neutrophils.

The platelet mobilization index (PMI) formula includes platelets and red blood cells. The PMI expresses the adaptative liberating capacity of platelets sequestered in splanchnic versus splenic reservoirs. An elevated PMI indicates acute stress when the effects of adrenaline are augmented, favoring splanchnic demargination. A low PMI reflects a relative insufficiency of adrenaline activity in adaptation [39].

The starter index is calculated as the ratio of LMI to PMI and expresses the relative predominance of glucose mobilization to start the adaptation response of glucagon relative to adrenaline. This index evaluates the autonomic vs. endocrine and splanchnic vs. splenic pathways involved in starting the adaptation response. A high starter index indicates that glucagon for glucose mobilization from the liver is more effective than the effect of adrenaline [31].

The adjusted genital ratio index is an indirect index calculated as the ratio of the genital ratio index to the starter index [39]. It evaluates the general global predominance of androgens in relationship to that of estrogens on tissue in acute adaptation.

The genito-thyroid (GT) ratio index is the ratio of neutrophils (%) to lymphocytes (%), which expresses the relative activity of the gonads in relationship to that of the thyroid [39]. A high GT ratio indicates a greater likelihood of the thyrotropic axis soliciting inflammation to aid in catabolism; therefore, it is an indicator of systemic inflammation and increased risk of morbidity. A low GT ratio indicates a weak adaptive response of the thyroid.

The thyroid-releasing hormone (TRH) reactivation index is calculated as the ratio of monocytes (%) to lymphocytes (%). A high TRH reactivation index indicates the degree of disadaptation of the organism and reflects the level of reactivation of the thyrotropic axis by the alpha-sympathetic system [39].

## 3. Results

### 3.1. Patient Characteristics and BoF Index Values

This study included 117 randomly selected kidney transplant recipients. Kidney biopsy and blood tests for BoF index calculation were performed at a median of 20 months after kidney transplantation, with 4.3 life years on immunosuppression overall (including immunosuppression used for previous transplants or autoimmune native kidney disease). Furthermore, 23.1% of patients had a second or third kidney transplant, and 37.9% of cases presented histological signs of allograft rejection. The main characteristics of our study population are provided in Table 1.

The median of each BoF index was calculated in the whole patient cohort, as presented in Table 2. Only a small group of individuals (12–34%) had BoF index values assumed as normal, according to the general population data. More than 50% of the patients had low adaptation, LMI, genital, and adjusted genital ratio indices, while other indices associated with catabolism or activation of the corticotropic axis (Cata-Ana, GT ratio, adrenal gland, and cortisol indices) were high in >50% of patients. In the subgroup of patients with normal kidney transplant histology (no rejection or other abnormalities), these tendencies were even more prominent: a higher percentage of patients had low adaptation, LMI, PMI, genital, and adjusted genital ratio indices, as well as high corticotropic axis index (see Table 3).

### 3.2. BoF Indices in Patients with and without Transplant Rejection

The patients were divided into two groups according to the histological diagnosis: patients without rejection on histology (non-rejectors: *n* = 70) and patients with transplant rejection (rejectors: *n* = 41). The index values were compared between two groups using the Mann–Whitney test (Table 4). The adaptation index was significantly higher in patients with biopsy-proven transplant rejection, while the ratio of cortisol to the adrenal gland index was significantly higher in the non-rejector group. The latter group included cases with normal histology and other-than-rejection histology abnormalities (e.g., recurrent glomerulonephritis and global glomerulosclerosis). The same significant results were observed when the indices were compared between the pure normal histology (*n* = 26) and rejection groups.

### 3.3. Cortisol and Arenal Gland Index Ratio

Cortisol should be interpreted together with the adrenal gland index; normally, this ratio should be ~3. In our cohort, only 22 (18.8%) patients had a normal cortisol/adrenal gland index ratio. In this group of normal ratio patients, only 9.1% had normal renal histology, 31.8% had transplant rejection, and 59.1% had other abnormalities on kidney biopsy. Furthermore, 13.6% of patients lost their transplant during the 12 months after graft biopsy, 50% of patients had a functioning graft 1 year after biopsy, and 36.4% of patients had less than 12 months of follow-up.

The patient group with an increased cortisol/adrenal gland index ratio had significantly fewer transplant rejection cases (31.9%) compared to the patients with a low cortisol/adrenal gland index ratio (60.9%; *p* < 0.05); however, the cortisol/adrenal gland index AUC for discriminating patients with transplant rejection was only 0.356 (CI 0.246–0.467). Looking at infectious complications, the group with the higher ratio had a higher average number of cytomegalovirus infection episodes per patient in the last 6 months before biopsy (0.29) compared to the group with the lower ratio (0.09), although this result was not significant (*p* > 0.05).

A total of 91% of patients with a low cortisol/adrenal gland index ratio received methylprednisolone (median dose: 8 mg), while 92% of patients with a high cortisol/adrenal gland index ratio received methylprednisolone (median dose: 6 mg). The median times (months) after transplantation in groups with low, normal, and high ratios were 36.00, 81.00, and 12.00, respectively, but significantly differed only between groups with normal and high ratios (*p* < 0.05).

### 3.4. Adaptation Index

**Figure 1:**
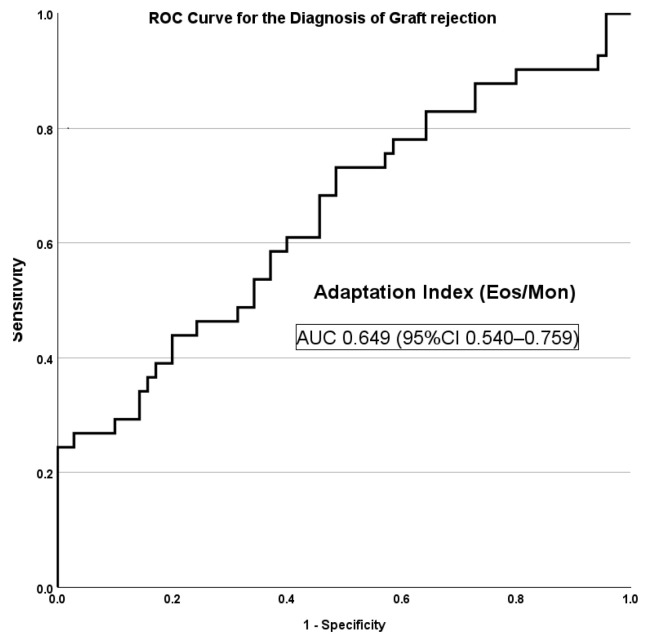
Adaptation index ROC curve for detecting biopsy-proven kidney transplant rejection. The AUC was 0.649 (95%CI 0.540–0.759).

Our data analysis revealed that more than two-thirds of the kidney transplant recipients had a decreased adaptation index, while patients with biopsy-proven transplant rejection had significantly higher adaptation index values. The ROC analysis of the adaptation index showed an AUC value of 0.649 (95%CI 0.540–0.759) for detecting biopsy-proven kidney transplant rejection (see Figure 1).

### 3.5. BoF Index Correlation with Clinical Parameters

Spearman correlation analysis revealed a significant negative correlation between the adaptation index and the tacrolimus level. The LMI and PMI correlated negatively with patient body mass index (BMI). A positive correlation was found between patient BMI and the GT ratio index. There were positive correlations between starter, genital, and adjusted genital ratio indices and graft survival. The serum creatinine concentration at the time of the biopsy correlated positively with the adrenal gland, TRH reactivation, Cata-Ana, and PMI indices and correlated negatively with the genital, adjusted genital ratio, and starter indices. The mycophenolate (MMF) dose correlated negatively with the PMI and positively with the starter index (Table 5). No significant correlations were observed between the considered indices and the steroid dose or cyclosporine concentration. Patients with tacrolimus levels below 5 ng/mL had a significantly higher median value of the adaptation index compared to patients with higher levels of tacrolimus (0.17 [0.11–0.39] vs. 0.10 [0.05–0.15]; *p* = 0.002); however, the other indices did not differ significantly between these groups.

### 3.6. BoF Indices and One-Year Graft Survival

The patients were divided into two groups: one group included patients with graft loss during the year after the biopsy (*n* = 18), while the second group included patients without graft loss during the year after the biopsy. It is worth noting that two patients were excluded, who died within 1–2 months after the biopsy due to cryptococcosis or pancreatic abscesses and had an infection at the time of the biopsy.

**Figure 2:**
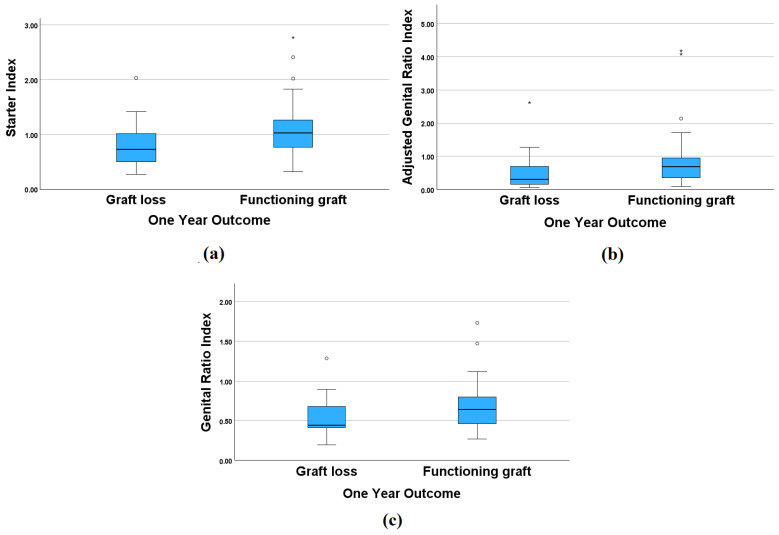
Starter (**a**), adjusted genital (**b**), and genital ratio (**c**) indices in patients with and without graft loss during follow-up 12 months after kidney biopsy. Mild outliers are marked with a circle (°), and extreme outliers are marked with an asterisk (*).

Non-parametric statistical tests (Mann–Whitney U test) were performed to compare different characteristics between the graft loss group and patients with a working graft (see Table 6). Only serum creatinine levels were significantly higher in the graft loss group; however, there was a tendency toward lower genital ratio, starter, and adjusted genital ratio in the graft loss group, although these differences were not significant (Figure 2).

## 4. Discussion

To the best of our knowledge, this is the first publication focused on endobiogenic indices in a kidney transplant recipient cohort. Although endobiogenic medicine uses many more indices and includes a holistic approach to the patient, including inspection, thorough anamnesis of the patient, and evaluation of the whole combination of indices (instead of drawing conclusions from one abnormal index), our aim was to reflect the tendencies of such indices in kidney transplant recipients receiving maintenance immunosuppressive therapy [21]. All indices in this paper were calculated from the complete blood count test, with differentials taken 0–7 days before kidney allograft biopsy, which was performed for various clinical indications.

The cortisol and adrenal gland indices represent the activity of the corticotropic axis. The cortisol index normally expresses the role of cortisol during adaptation; however, patients after kidney transplantation who regularly take steroids for immunosuppression are likely to have an increased cortisol index and an increased cortisol/adrenal gland ratio. In our cohort, 91% of patients with a low cortisol index were taking steroids, while more than 60% of patients with a low index ratio had biopsy-proven transplant rejection at the time of measurement. This finding suggests that some recipients with low values of this ratio index may show a state of under-immunosuppression. In contrast, there was a tendency toward higher numbers of CMV infections and a lower rate of transplant rejection in patients with a high cortisol/adrenal gland ratio, indicating a state of over-immunosuppression, despite receiving a lower dose of steroids compared to the group with a low cortisol/adrenal gland index ratio. Therefore, this index ratio might be helpful in identifying individual patients presenting over- or under-immunosuppression for the guidance of immunosuppressive agent dosing; however, more data are needed for validation.

Our data analysis showed that more than two-thirds of kidney transplant recipients had a lower-than-normal adaptation index, which is not surprising as this index is calculated as the ratio of eosinophils to monocytes and, in transplanted patients, oral immunosuppressive therapy with corticosteroids may lower eosinophil levels [40]. Our results revealed that patients experiencing allograft rejection had significantly higher adaptation index values, indicating lower glucocorticoid activity. Moreover, there are published data indicating that eosinophilia often precedes T-cell-mediated transplant rejection, as well as the successful use of corticosteroids for managing this type of rejection through the suppression of eosinophils [41,42].

More than half of the patients had decreased adaptation, LMI, genital, and adjusted genital ratio indices. According to the theory of endobiogeny, these data indicate that kidney transplant recipients tend to have low ACTH activity due to the inhibition caused by high cortisol activity associated with steroid use. A low LMI suggests a dysfunctional response to aggression and a tendency to solicit white blood cells from the perihepatic circulation more than from the splanchnic circulation. It is calculated as the ratio of platelets, neutrophils, and hemoglobin to white blood cells, and in our cohort of kidney transplant recipient patients, this ratio was decreased in more than half of the patients. Moreover, the indices associated with catabolism and activation of the corticotropic axis were increased, indicating an effect of steroid catabolic activity.

Higher tacrolimus levels were associated with lower patient adaptation. This can be explained by the ability of tacrolimus to reduce eosinophil levels by promoting eosinophil apoptosis [43]; however, tacrolimus does not strongly affect monocyte function to a clinically relevant degree and only changes macrophage polarization [44].

Patients with a higher BMI had lower PMI and LMI values, which can be explained by the higher platelet count in patients with an increased BMI, as mentioned in several papers [45,46,47], and a positive significant correlation between BMI and platelet count was observed in our data. Notably, platelets are present in the denominator of both index formulas. Higher BMI was associated with a higher GT index, which is the ratio of neutrophils to lymphocytes, and data on neutrophilia and lymphocytopenia in obese patients have been previously reported [48,49,50]. According to the theory of endobiogeny, an elevated GT ratio indicates efficient thyroid activity and the likelihood of the thyrotropic axis to solicit inflammation to aid in catabolism [31].

A tendency of longer graft survival was observed in patients with higher starter, genital, and adjusted genital ratios. The genital ratio increases with higher red blood cells and reflects the dominant activity of androgens over estrogens, according to the theory of endobiogeny. However, longer graft survival is closely associated with better functioning of the graft, and on the other hand, anemia is a sign of kidney failure [51]. Therefore, higher red blood cells and a higher genital ratio are linked to better functioning of the graft and, thus, longer graft survival.

The negative correlation between the MMF dose and PMI can be explained by the effect of MMF on bone marrow function; in particular, it suppresses the production of red blood cells more than platelets [52,53]. The PMI is in the denominator of the starter index formula; so, the latter index is positively correlated with the MMF dose. In endobiogenic medicine, an increased starter index is interpreted as increased glucose mobilization from liver glucagon in response to stress and the need for adaptation [22].

One of the limitations is that the study patients formed a very heterogenic group of kidney transplant recipients with a very different duration of overall immunosuppression, with the time after transplantation ranging from 0 to 28 years. In other words, the patients may not have been matched regarding variables that can affect clinical outcomes, including graft loss and duration of graft survival. Moreover, the allograft histology was very heterogenous, including normal histology as well as transplant rejection (both antibody-mediated and T-cell-mediated), recurrent glomerulopathy, polyoma virus infection, global glomerulosclerosis, and so on. It would be rational to analyze BoF indices in larger groups of patients with each histological diagnosis. Moreover, we did not have data about patient comorbidities such as diabetes, thyroid diseases, and medications used for their therapy, which could affect the BoF index results.

Despite these limitations, we provide the first overview of endobiogenic indices in a very specific patient population and connect the interpretation of these indices from traditional and endobiogenic medicine points of view. By increasingly applying an individualized approach to patients in clinical practice, the principles of endobiogenic medicine could complement traditional medicine, especially when making decisions about the patient’s treatment, while assessing the risk of complications. BoF indices provide additional knowledge about the patient’s endocrine system activity, metabolism, and other ongoing processes; however, so far, these indices have been studied and applied mainly in the general population. Chronic kidney disease and chronic immunosuppression significantly alter physiological processes; so, the interpretation of BoF indices applied to the general population may not be appropriate for patients after kidney transplantation. As such, more scientific evidence is needed to apply the principles of endobiogenic medicine to this patient population. This article provides data about BoF values in patients with normal graft histology, as well as the correlation between indices and graft rejection and over-immunosuppressive conditions.

## 5. Conclusions

Most of the kidney transplant recipients had abnormal BoF index values, reflecting increased corticotropic effects on their cells. This was even more prominent in the subgroup of patients with normal transplant histology. Therefore, BoF indices should be interpreted with caution in kidney transplant recipient populations due to the corticotropic effects of maintenance immunosuppressive therapy. Among all the calculated indices, only the adaptation index discriminated patients with biopsy-proven transplant rejection from those without it. As such, this index may be used in addition to other biomarkers when kidney transplant rejection is suspected.

## Figures and Tables

**Table 1:** Patient characteristics (*n* = 117).

Gender	Male, *n* (%)	74 (63.2)
	Female, *n* %	43 (36.8)
Age	Average age, years	43 ± 13
eGFR at biopsy *	mL/min/1.73 m^2^	39 ± 18
Biopsy time after transplantation	Median months [IQR]	20.0 [6–96]
Number of transplantations	First transplant: *n* (%) of patients	90 (76.9)
	Second transplant: *n* (%) of patients	22 (18.8)
	Third transplant: *n* (%) of patients	5 (4.3)
Total life years on immunosuppression	Median [IQR]	4.3 [1.0–11.0]
Hemoglobin at biopsy	g/L	115.8 ± 19.8
BMI	kg/m^2^	24.7 ± 4.8
Serum urea at biopsy	mmol/L	20.4 ± 12.6
Biopsy result (% of cases)	Normal histology, %	22.2
	ABMR, %	21.4
	TCMR, %	7.7
	Mixed rejection, %	8.8
	BK virus nephropathy, %	4.4
	Other abnormalities, %	35.9
Immunosuppression	Tacrolimus, %	66.7
	Cyclosporine, %	25.6
	Mycophenolate, %	93.2
	Methylprednisolone, %	92.3
	Azathioprine, %	2.6
	Sirolimus, %	7.7
Serum levels of immunosuppressive agent	Tacrolimus, ng/mL	6.57 ± 3.78
	Cyclosporine, ng/mL (before dose)	84.74 ± 27.15
	Sirolimus, ng/mL	0.83 ± 0.65

BMI—body mass index, ABMR—antibody-mediated rejection, and TCMR—T-cell-mediated rejection. * eGFR was estimated using the CKD EPI 2021 formula.

**Table 2:** BoF index median values in a cohort of 117 kidney transplant recipients and the percentage of patients with normal, lower-than-normal, or higher-than-normal index values. BoF index formulas are presented in the table.

Index	Formula	Normal Limits	Median	% of Patients with a LOW Index Value	% of Patients with a NORMAL Index Value	% of Patients with a HIGH Index Value
Adaptation	Eos/Mon	0.25–0.50	0.11 [0.07–0.21]	76.9	12.0	11.1
LMI	(PLT × Neu × HgB)/(30,000 × WBCs)	0.85–1.15	0.76 [0.60–0.92]	59.8	32.5	7.7
PMI	PLT/(60 × RBCs)	0.85–1.15	0.85 [0.66–1.11]	47.9	34.2	17.9
Starter	LMI/PMI	0.85–1.15	0.90 [0.67–1.18]	42.7	30.8	26.5
Cata-Ana	GT ratio/(Genital ratio × Starter index)	1.80–3.0	4.99 [2.92–8.00]	6.8	23.9	69.2
GT ratio	Neu/Lymph	1.50–2.50	3.07 [2.19–4.21]	10.3	30.8	59.0
Genital ratio	RBCs/WBCs	0.80–0.95	0.63 [0.45–0.76]	76.1	16.2	7.7
Adjusted Genital ratio	Genital ratio index × Starter index	0.85–1.05	0.58 [0.30–0.86]	70.1	17.1	12.8
Adrenal Gland	GT ratio/(Genital ratio)^2^	2.70–3.30	7.61 [4.01–16.37]	12.0	12.8	75.2
Cortisol	Cata-ana index/Adaptation index	3–7	37.42 [16.42–111.53]	2.6	14.5	82.9
Cortisol/Adrenal Gland Ratio		3		19.7	18.8	61.5
TRH reactivation	Mon/Lymph	0.05–0.25	0.44 [0.29–0.59]	1.7	15.4	82.9

Eos—eosinophils (in %), Lymph—lymphocytes (in %), Mon—monocytes (in %), Neu—neutrophils (in %), PLT—platelets (in units/mm^3^, i.e., 185,000), RBCs—red blood cells (in thousands/mm^3^, i.e., 4400), and WBCs—white blood cells (in units/mm^3^, i.e., 6100).

**Table 3:** Patients with normal transplant histology: percentage of patients with low, normal, and high values of each BoF index.

Index	% of Patients with a LOW Index Value	% of Patients with a NORMAL Index Value	% of Patients with a HIGH Index Value
Adaptation	96.2	0	3.8
LMI	65.4	30.8	3.8
PMI	50.0	38.5	11.5
Starter	38.5	26.9	34.6
Cata-Ana	11.5	19.2	69.2
GT ratio	15.4	34.6	50.0
Genital ratio	84.6	7.7	7.7
Adjusted Genital ratio	73.1	15.4	11.5
Adrenal Gland	11.5	3.8	84.6
Cortisol	3.8	7.7	88.5
TRH reactivation	0	7.7	92.3

**Table 4:** Median [IQR] BoF index values in patients with biopsy-proven transplant rejection and patients with no rejection (normal histology or other histological abnormalities such as global glomerulosclerosis or recurrent glomerulonephritis).

Index	Rejectors (Median [IQR])	Non-Rejectors (Median [IQR])	*p*-Value
Adaptation	0.15 [0.09–0.43]	0.10 [0.05–0.18]	0.009 **
LMI	0.80 [0.61–0.96]	0.71 [0.58–0.90]	0.172
PMI	0.79 [0.64–1.09]	0.87 [0.66–1.12]	0.621
Starter	0.98 [0.70–1.27]	0.87 [0.64–1.15]	0.236
Cata-Ana	4.5 [2.91–8.29]	5.02 [3.01–8.01]	0.779
GT ratio	2.98 [2.23–4.06]	0.62 [0.44–0.72]	0.908
Cortisol	29.54 [10.21–87.94]	47.39 [18.50–126.40]	0.157
Genital ratio	0.67 [0.45–0.80]	0.62 [0.44–0.72]	0.460
Adrenal gland	6.67 [3.27–17.61]	8.21 [4.30–16.65]	0.467
Adjusted Genital ratio	0.69 [0.31–0.97]	0.53 [0.30–0.82]	0.258
Cortisol/Adrenal gland ratio	3.77 [1.86–6.66]	5.71 [3.41–9.40]	0.013 *
TRH reactivation	0.41 [0.28–0.56]	0.47 [0.30–0.65]	0.259

**. Significance at the 0.01 level. *. Significance at the 0.05 level.

**Table 5:** Spearman correlation analysis of BoF indices and patient clinical data.

Index	BMI	Graft Survival (Months Until Dialysis)	Serum Creatinine Level at the Time of the Biopsy	Tacrolimus Level (ng/mL)	MMF Dose
Adaptation	−0.062	0.196	0.148	−0.270 *	−0.049
LMI	−0.194 *	0.265	−0.062	0.026	0.624
PMI	−0.337 **	−0.245	0.218 *	−0.063	−0.199 *
Starter	0.156	0.401 *	−0.241 *	0.153	0.221 *
Cata-Ana	0.152	−0.254	0.323 **	−0.072	−0.057
GT ratio	0.204 *	−0.283	0.327 **	−0.007	−0.013
Genital ratio	0.018	0.377 *	−0.218 *	0.122	0.129
Adjusted Genital ratio	0.092	0.387 *	−0.235 *	0.138	0.174
Adrenal Gland	0.108	−0.276	0.276 **	−0.082	−0.060
Cortisol	0.142	−0.074	0.056	0.169	0.066
Cortisol/Adrenal Gland Ratio	−0.046	−0.041	−0.144	0.301	0.135
TRH reactivation	0.140	−0.085	0.250 **	−0.006	−0.090

** Correlation is significant at the 0.01 level. * Correlation is significant at the 0.05 level.

**Table 6:** Characteristics and BoF indices in patients with and without graft loss during the follow-up 12 months after kidney biopsy.

Characteristics	Graft Loss (Median [IQR])	No Graft Loss (Median [IQR])	*p*-Value
Patient age	31.50 [28.00–56.50]	43.50 [31.2–53.75]	0.222
BMI	21.92 [20.62–26.00]	24.62 [20.82–28.69]	0.431
Creatinine at biopsy, µmol/L	430.00 [294.50–472.50]	155.00 [121.00–231.00]	<0.001 *
Creatinine at 1 month after biopsy, µmol/L	235.73 [148.00–314.00]	216.18 [110.75–247.50]	0.042 *
Creatinine 3 months after biopsy, µmol/L	323.50 [221.25–386.25]	128.00 [108.00–175.00]	0.043 *
Creatinine 6 months after biopsy, µmol/L	385.50 [313.5–506.5]	153.00 [108.50–218.00]	0.023 *
Biopsy time after transplantation	48.50 [11.50–107.5]	16.00 [8.00–73.50]	0.161
C-reactive protein	1.03 [0.29–8.25]	1.70 [0.90–3.77]	0.932
Tacrolimus level, ng/mL	5.00 [3.68–7.68]	5.90 [4.00–7.70]	0.780
Mycophenolate dose, g	1.5 [1.0–2.0]	2.0 [1.4–2.0]	0.166
Steroid dose, mg	8.00 [4.00–9.00]	4.00 [4.00–8.00]	0.119
BoF Indices:			
Adaptation	0.11 [0.04–0.28]	0.12 [0.07–0.20]	0.838
Cata-Ana	6.98 [3.08–19.96]	4.80 [2.57–6.78]	0.735
GT ratio	3.94 [2.32–7.56]	2.91 [2.08–3.62]	0.378
Cortisol	58.24 [9.87–155.48]	39.25 [14.51–95.47]	0.776
Genital ratio	0.44 [0.39–0.69]	0.64 [0.45–0.80]	0.067
Adrenal gland	12.44 [4.50–41.89]	7.48 [3.17–15.33]	0.378
TRH reactivation	0.44 [0.19–0.72]	0.42 [0.30–0.52]	0.838
LMI	0.59 [0.50–0.88]	0.80 [0.62–0.97]	0.197
PMI	0.90 [0.76–1.11]	0.75 [0.63–1.04]	0.154
Starter	0.73 [0.49–1.03]	1.03 [0.76–1.27]	0.067
Adjusted genital ratio	0.31 [0.16–0.72]	0.69 [0.35–0.96]	0.067

* Significance at the 0.05 level.

## Data Availability

The raw data supporting the conclusions of this article will be made available by the author upon request.

## References

[1] Chaudhry D., Chaudhry A., Peracha J., Sharif A. (2022). Survival for Waitlisted Kidney Failure Patients Receiving Transplantation versus Remaining on Waiting List: Systematic Review and Meta-Analysis. BMJ.

[2] Augustine J. (2018). Kidney Transplant: New Opportunities and Challenges. Cleve. Clin. J. Med..

[3] Vamenta-Morris H., Keith D.S. (2015). Chronic Maintenance Immunosuppression in Renal Transplantation: The Unrealized Goal of Improved Long-Term Outcomes. Minerva Urol. Nefrol..

[4] Van Den Born J.C., Meziyerh S., Vart P., Bakker S.J.L., Berger S.P., Florquin S., De Fijter J.W., Gomes-Neto A.W., Idu M.M., Pol R.A. (2024). Comparison of 2 Immunosuppression Minimization Strategies in Kidney Transplantation: The ALLEGRO Trial. Transplantation.

[5] Van Sandwijk M.S., De Vries A.P.J., Bakker S.J.L., Ten Berge I.J.M., Berger S.P., Bouatou Y.R., De Fijter J.W., Florquin S., Homan Van Der Heide J.J., Idu M.M. (2018). Early Steroid Withdrawal Compared With Standard Immunosuppression in Kidney Transplantation—Interim Analysis of the Amsterdam-Leiden-Groningen Randomized Controlled Trial. Transplant. Direct.

[6] Cheung C.Y., Tang S.C.W. (2019). An Update on Cancer after Kidney Transplantation. Nephrol. Dial. Transplant..

[7] Borriello M., Ingrosso D., Perna A.F., Lombardi A., Maggi P., Altucci L., Caraglia M. (2022). BK Virus Infection and BK-Virus-Associated Nephropathy in Renal Transplant Recipients. Genes.

[8] Rostaing L., Wéclawiak H., Mengelle C., Kamar N. (2011). Viral Infections after Kidney Transplantation. Minerva Urol. Nefrol..

[9] Agrawal A., Ison M.G., Danziger-Isakov L. (2022). Long-Term Infectious Complications of Kidney Transplantation. CJASN.

[10] Azadi S., Azarpira N., Roozbeh J., Ezzatzadegan-Jahromi S., Raees-Jalali G.A., Foroughinia F., Karimzadeh I. (2023). Genetic Polymorphisms of Glucocorticoid Receptor and Their Association with New-Onset Diabetes Mellitus in Kidney Transplant Recipients. Gene.

[11] Faddoul G., Nadkarni G.N., Bridges N.D., Goebel J., Hricik D.E., Formica R., Menon M.C., Morrison Y., Murphy B., Newell K. (2018). Analysis of Biomarkers Within the Initial 2 Years Posttransplant and 5-Year Kidney Transplant Outcomes: Results From Clinical Trials in Organ Transplantation-17. Transplantation.

[12] Loupy A., Haas M., Roufosse C., Naesens M., Adam B., Afrouzian M., Akalin E., Alachkar N., Bagnasco S., Becker J.U. (2020). The Banff 2019 Kidney Meeting Report (I): Updates on and Clarification of Criteria for T Cell- and Antibody-Mediated Rejection. Am. J. Transpl..

[13] Haas M., Loupy A., Lefaucheur C., Roufosse C., Glotz D., Seron D., Nankivell B.J., Halloran P.F., Colvin R.B., Akalin E. (2018). The Banff 2017 Kidney Meeting Report: Revised Diagnostic Criteria for Chronic Active T Cell-Mediated Rejection, Antibody-Mediated Rejection, and Prospects for Integrative Endpoints for next-Generation Clinical Trials. Am. J. Transpl..

[14] Mayer K.A., Omic H., Weseslindtner L., Doberer K., Reindl-Schwaighofer R., Viard T., Tillgren A., Haindl S., Casas S., Eskandary F. (2022). Levels of Donor-Derived Cell-Free DNA and Chemokines in BK Polyomavirus-Associated Nephropathy. Clin. Transpl..

[15] Arnau A., Benito-Hernández A., Ramos-Barrón M.A., García-Unzueta M.T., Gómez-Román J.J., Gómez-Ortega J.M., López-Hoyos M., San Segundo D., Ruiz J.C., Rodrigo E. (2021). Urinary C-X-C Motif Chemokine 10 Is Related to Acute Graft Lesions Secondary to T Cell- and Antibody-Mediated Damage. Ann. Transpl..

[16] Fahmy N.M., Yamani M.H., Starling R.C., Ratliff N.B., Young J.B., McCarthy P.M., Feng J., Novick A.C., Fairchild R.L. (2003). Chemokine and Chemokine Receptor Gene Expression Indicates Acute Rejection of Human Cardiac Transplants. Transplantation.

[17] Hirt-Minkowski P., Handschin J., Stampf S., Hopfer H., Menter T., Senn L., Hönger G., Wehmeier C., Amico P., Steiger J. (2023). Randomized Trial to Assess the Clinical Utility of Renal Allograft Monitoring by Urine CXCL10 Chemokine. J. Am. Soc. Nephrol..

[18] Zhang R. (2018). Donor-Specific Antibodies in Kidney Transplant Recipients. CJASN.

[19] Cheung C.Y., Tang S.C.W. (2022). Personalized Immunosuppression after Kidney Transplantation. Nephrology.

[20] Doberer K., Schiemann M., Strassl R., Haupenthal F., Dermuth F., Görzer I., Eskandary F., Reindl-Schwaighofer R., Kikić Ž., Puchhammer-Stöckl E. (2020). Torque Teno Virus for Risk Stratification of Graft Rejection and Infection in Kidney Transplant Recipients—A Prospective Observational Trial. Am. J. Transpl..

[21] Lapraz J.-C., Hedayat K.M. (2013). Endobiogeny: A Global Approach to Systems Biology (Part 1 of 2). Glob. Adv. Health Med..

[22] Lapraz J.-C., Hedayat K.M., Pauly P. (2013). Endobiogeny: A Global Approach to Systems Biology (Part 2 of 2). Glob. Adv. Health Med..

[23] Hedayat K.M. (2020). The Theory of Endobiogeny: Biological Modeling Using Downstream Physiologic Output as Inference of Upstream Global System Regulation. J. Complex. Health Sci..

[24] Haller M.C., Kainz A., Baer H., Oberbauer R. (2017). Dialysis Vintage and Outcomes after Kidney Transplantation: A Retrospective Cohort Study. CJASN.

[25] Stangou M.J., Fylaktou A., Ivanova-Shivarova M.I., Theodorou I. (2022). Editorial: Immunosenescence and Immunoexhaustion in Chronic Kidney Disease and Renal Transplantation. Front. Med..

[26] Salminen A. (2021). Immunosuppressive Network Promotes Immunosenescence Associated with Aging and Chronic Inflammatory Conditions. J. Mol. Med..

[27] Stavropoulou E., Kantartzi K., Tsigalou C., Aftzoglou K., Voidarou C., Konstantinidis T., Chifiriuc M.C., Thodis E., Bezirtzoglou E. (2021). Microbiome, Immunosenescence, and Chronic Kidney Disease. Front. Med..

[28] Hedayat K.M., Chalvet D., Yang M., Golshan S., Allix-Beguec C., Beneteaud S., Schmit T. (2022). Evolution of Modeled Cortisol Is Prognostic of Death in Hospitalized Patients With COVID-19 Syndrome. Front. Med..

[29] Braukyliene R., Hedayat K., Zajanckauskiene L., Jurenas M., Unikas R., Aldujeli A., Petrokas O., Zabiela V., Steponaviciute R., Vitkauskiene A. (2021). Prognostic Value of Cortisol Index of Endobiogeny in Acute Myocardial Infarction Patients. Medicina.

[30] Hedayat K., Shuff B.M., Lapraz J.-C., Barsotti T., Golshan S., Hong S., Greenberg B., Mills P.J. (2017). Genito-Thyroid Index: A Global Systems Approach to the Neutrophil-to-Lymphocyte Ratio According to the Theory of Endobiogeny Applied to Ambulatory Patients with Chronic Heart Failure. J. Cardiol. Clin. Res..

[31] Hedayat K.M., Lapraz J.-C. (2019). A New Approach to Biological Modeling: Introduction to the Biology of Functions. The Theory of Endobiogeny.

[32] Beishuizen A., Vermes I. (1999). Relative Eosinophilia (Thorn Test) as a Bioassay to Judge the Clinical Relevance of Cortisol Values during Severe Stress. J. Clin. Endocrinol. Metab..

[33] Pelekanou V., Kampa M., Kiagiadaki F., Deli A., Theodoropoulos P., Agrogiannis G., Patsouris E., Tsapis A., Castanas E., Notas G. (2016). Estrogen Anti-Inflammatory Activity on Human Monocytes Is Mediated through Cross-Talk between Estrogen Receptor ERα36 and GPR30/GPER1. J. Leukoc. Biol..

[34] Okamoto M., Suzuki T., Mizukami Y., Ikeda T. (2017). The Membrane-type Estrogen Receptor G-protein-coupled Estrogen Receptor Suppresses Lipopolysaccharide-induced Interleukin 6 via Inhibition of Nuclear Factor-kappa B Pathway in Murine Macrophage Cells. Anim. Sci. J..

[35] Garrod O. (1958). The Pharmacology of Cortisone, Cortisol (Hydrocortisone) and Their New Analogues. Postgrad. Med. J..

[36] Rinehart J.J., Balcerzak S.P., Sagone A.L., LoBuglio A.F. (1974). Effects of Corticosteroids on Human Monocyte Function. J. Clin. Investig..

[37] Sabag N., Castrillón M.A., Tchernitchin A. (1978). Cortisol-Induced Migration of Eosinophil Leukocytes to Lymphoid Organs. Experientia.

[38] Thorn G.W. (1948). A Test for Adrenal Cortical Insufficiency: The Response to Pituitary Andrenocorticotropic Hormone. JAMA.

[39] Hedayat K.M., Lapraz J.-C. (2019). The Theory of Endobiogeny. Volume 1, Global Systems Thinking and Biological Modeling for Clinical Medicine.

[40] Ortega H., Llanos J.-P., Lafeuille M.-H., Duh M.S., Germain G., Lejeune D., Sama S., Bell C., Hahn B. (2019). Effects of Systemic Corticosteroids on Blood Eosinophil Counts in Asthma: Real-World Data. J. Asthma.

[41] Lautenschlager I., Willebrand E.V., Häyry P. (1985). Blood eosinophilia, steroids, and rejection. Transplantation.

[42] Almirall J., Campistol J.M., Sole M., Andreu J., Revert L. (1993). Blood and Graft Eosinophilia as a Rejection Index in Kidney Transplant. Nephron.

[43] Kandikattu H.K., Venkateshaiah S.U., Verma A.K., Mishra A. (2021). Tacrolimus (FK506) Treatment Protects Allergen-, IL-5- and IL-13-induced Mucosal Eosinophilia. Immunology.

[44] Kannegieter N.M., Hesselink D.A., Dieterich M., Kraaijeveld R., Rowshani A.T., Leenen P.J.M., Baan C.C. (2017). The Effect of Tacrolimus and Mycophenolic Acid on CD14+ Monocyte Activation and Function. PLoS ONE.

[45] Purdy J.C., Shatzel J.J. (2021). The Hematologic Consequences of Obesity. Eur. J. Haematol..

[46] Samocha-Bonet D., Justo D., Rogowski O., Saar N., Abu-Abeid S., Shenkerman G., Shapira I., Berliner S., Tomer A. (2008). Platelet Counts and Platelet Activation Markers in Obese Subjects. Mediat. Inflamm..

[47] Konstantinides S., Schäfer K., Koschnick S., Loskutoff D.J. (2001). Leptin-Dependent Platelet Aggregation and Arterial Thrombosis Suggests a Mechanism for Atherothrombotic Disease in Obesity. J. Clin. Investig..

[48] Aydın M. (2015). Neutrophil Lymphocyte Ratio in Obese Adolescents. North. Clin. Istanb..

[49] Fang Q., Tong Y.-W., Wang G., Zhang N., Chen W.-G., Li Y.-F., Shen K.-W., Wu B.-W., Chen X.-S. (2018). Neutrophil-to-Lymphocyte Ratio, Obesity, and Breast Cancer Risk in Chinese Population. Medicine.

[50] Kain V., Van Der Pol W., Mariappan N., Ahmad A., Eipers P., Gibson D.L., Gladine C., Vigor C., Durand T., Morrow C. (2019). Obesogenic Diet in Aging Mice Disrupts Gut Microbe Composition and Alters Neutrophi:Lymphocyte Ratio, Leading to Inflamed Milieu in Acute Heart Failure. FASEB J..

[51] Iwamoto H., Nakamura Y., Konno O., Hama K., Yokoyama T., Kihara Y., Kawachi S., Shimazu M. (2014). Correlation Between Post Kidney Transplant Anemia and Kidney Graft Function. Transplant. Proc..

[52] Van Besouw N.M., Van Der Mast B.J., Smak Gregoor P.J.H., Hesse C.J., IJzermans J.N.M., Van Gelder T., Weimar W. (1999). Effect of Mycophenolate Mofetil on Erythropoiesis in Stable Renal Transplant Patients Is Correlated with Mycophenolic Acid Trough Levels. Nephrol. Dial. Transplant..

[53] Pile T., Kieswich J., Harwood S., Yaqoob M.M. (2011). A Possible Explanation for Anemia in Patients Treated With Mycophenolic Acid. Transplantation.

